# Dental Files

**Published:** 1850-10

**Authors:** 


*Dental Files.*—Until within the last few years, the dentists of the United States were wholly dependent on Stub's manufactory in England for all the files they used. In fact, the superior excellence of the files of this manufactory, for the teeth, has been universally acknowledged. The quality of Stub's separating files for the front teeth have never been surpassed, and, until quite recently, we supposed him the only manufacrurer in the world who made a file suitable for the teeth, but we have lately used some molar files, and also some files of Dr. Townsend's invention, manufactured by Mr. Murphy, of Philadelphia, equal in quality, to any we have ever used. We mention this for the information of those who may not be aware of the fact that dental files are manufactured in this country.

